# The oncological and surgical safety of robot-assisted surgery in colorectal cancer: outcomes of a longitudinal prospective cohort study

**DOI:** 10.1007/s00464-018-06653-2

**Published:** 2019-01-28

**Authors:** F. Polat, L. H. Willems, K. Dogan, C. Rosman

**Affiliations:** 1 Department of surgery, Canisius Wilhelmina Hospital, Nijmegen, The Netherlands; 2 Department of gastro-intestinal and oncologic surgery, Radboud University Medical Centre, Nijmegen, The Netherlands

**Keywords:** Robotic surgery, Laparoscopic surgery, Colorectal cancer, DaVinci Xi, Oncological safety

## Abstract

**Background:**

Colorectal cancer is one of the most common cancers worldwide. Laparoscopic colorectal surgery (LCRS) is a frequently used modality. A new development in minimally invasive surgery is robot-assisted colorectal surgery (RACRS).

**Methods:**

Prospectively collected data of 378 consecutive patients who underwent RACRS or LCRS for stage I–III colorectal cancer from Dec 2014 to Oct 2017 were analyzed. Primary outcome was oncological outcome (radical margins, number of retrieved lymph nodes, locoregional recurrence). Secondary outcomes were distant metastases, overall and disease-free survival, operation time, conversion, length of hospital stay, and intra- and post-operative complications.

**Results:**

206 RACRS (129 colon and 77 rectal) and 172 LCRS (138 colon and 34 rectal) procedures were included. Baseline characteristics were similar. Overall median follow-up time was 15 months (0.2–36). Oncological outcome was similar. In colon cancer, radical margins were achieved in 99.3% in RACRS group versus 98.6% in LCRS group (*p* = 0.60), the average number of harvested lymph nodes was 16 ± 6 versus 18 ± 7 (*p* = 0.16), and locoregional recurrence rate in 24 months was 3.8% vs 3.8% (*p* = 0.99), respectively. In rectal cancer, radical margins were achieved in 89.6% in RACRS group versus 94.3% in LCRS group (*p* = 0.42), the average number of harvested lymph nodes was 16 ± 8 versus 15 ± 4 (*p* = 0.51), and locoregional recurrence rate in 24 months was 9.5 versus 5.6% (*p* = 0.42), respectively. Incidence of metastasis, survival rates, operation time, length of hospital stay, and number of severe post-operative complications measured by Clavien–Dindo scores did not differ between RACRS and LCRS groups. Conversion and intra-operative complication rates were significantly lower in the RACRS group as compared to the LCRS group (3% vs 9%, *p* = 0.008 and 2% vs 8%, *p* = 0.003, respectively).

**Conclusion:**

RACRS is safe in the treatment of patients with stage I–III colorectal cancer. Oncological outcome did not differ between RACRS and LCRS groups. RACRS had lower conversion and intra-operative complication rates.

Colorectal cancer is one of the most common cancers in the world, the third most common in men, and the second most common in women. Yearly, approximately 700,000 patients with colorectal cancer die worldwide [1]. Introduction of the screening for colorectal cancer in the Netherlands over the past few years is expected to result in a higher percentage of early-stage localized colorectal cancer amenable for surgical resection with curative intent [2].

Both open and laparoscopic surgeries have been proven to be safe and laparoscopic resections are associated with several short-term advantages [3]. Locoregional recurrence of laparoscopic colon resections is similar to open surgery [4]. For rectal cancer, oncological outcome parameters are recently evaluated in four randomized controlled trials. Two of them demonstrated comparable oncological outcomes [5, 6]. In the other two, non-inferiority of laparoscopic surgery compared with open surgery was not established for pathologic outcome [7, 8].

A new development in minimally invasive surgery is robot-assisted colorectal surgery (RACRS). Potential benefits of RACRS include improved visualization due to a three-dimensional and magnified image with stable camera platform, advanced dexterity of instruments, and the possibility of the surgeon solely controlling the camera and assist arm, allowing maximal control. These benefits may improve clinical and oncological outcomes in colorectal surgery. Current available literature presumes RARCS to be safe with comparable short-term results as compared to laparoscopic colorectal surgery (LCRS) [9–12]. However, more importantly, sufficient research about oncological outcome is lacking.

Expectations for the future are that RACRS will overcome the possible shortcomings of conventional techniques, improving oncological outcomes and reducing complication rates. Therefore, the aim of this study is to enhance knowledge of the oncological and surgical safety of RACRS as a treatment for colorectal cancer.

## Materials and methods

### Study design

A single-center, longitudinal, prospective cohort study was performed. All procedures have been performed in the Canisius Wilhelmina Hospital (CWH), Nijmegen, the Netherlands. CWH is a large-volume teaching hospital for laparoscopic and robot-assisted surgery. All laparoscopic procedures were performed by dedicated laparoscopic surgeons, and surgeons performing RACRS were certificated for the daVinci robot system (Intuitive Surgical, Sunnyvale, California, United States). The study was started with the acquisition of the daVinci robot system by the CWH and contains an implementation phase. Peri-operative, pathological, and clinical follow-up data were collected.

The study protocol was approved by the local board of ethics of the Canisius Wilhelmina Hospital (CWH).

### Outcome parameters

Data on patient, tumor, operative characteristics (duration of operation, intra-operative complications, conversion), neoadjuvant treatment, post-operative complications (short and long term), pathology results (resection margin, number of retrieved lymph nodes), length of hospital stay, readmissions, post-operative mortality (30 days), locoregional recurrence, distant metastases, overall survival (OS), and disease-free survival (DFS) were recorded and analyzed.

Resection margins and number of retrieved lymph nodes were evaluated by examining pathologist reports, consisting of the microscopically assessment of pathology specimens. Circumferential resection margin (CRM) was defined positive if malignant cells were found at microscopy at 1 mm or less from the CRM. Proximal and distal resection margins were defined positive if malignant cells of the outermost part of the tumor were found at microscopy at the cutting edge of the tissue.

Locoregional recurrence of disease and distant metastases was diagnosed by pathological confirmation after resection or biopsy, or by radiological detection of lesions that increased in size over time. Locoregional recurrence of disease was defined as reappearance of cancer at the primary cancer site or in lymph nodes. Distant metastases were defined as malignant cells that have spread to distant organs, such as liver, lung, or peritoneum.

Complications were classified using the Clavien–Dindo classification [13]. Severe complications were defined as corresponding to grade 2–5 of the Clavien–Dindo scale. The date of report of a complication was considered as the date the complication occurred. Anastomotic leakage was defined as intestinal wall defect at the anastomotic site with communication between the intraluminal and extraluminal compartments, confirmed by radiological examination, endoscopy, or re-operation.

Laparotomy for any other reason than specimen extraction from the abdomen was considered as conversion. Operation time was defined as the total time from patient in to patient out of the operating room. Incision time was defined as total time from incision to skin closure.

### Population

Consecutive patients that underwent RACRS or LCRS for primary resectable clinical stage I–III (T1-3N0-2M0) colorectal cancer between December 2014 and October 2017 were selected for a retrospective analysis of a prospectively collected database. Exclusion criteria were patients who were not suitable for minimal invasive approach (i.e., patients with a history of major gastro-intestinal surgery by laparotomy or patients with major cardiopulmonary history with expected anesthesiological complaints in minimal invasive surgery), emergency surgery, or operation due to locoregional recurrence. Whether a patient could be distributed to RACRS or LCRS was solely dependent on the operational schedule and the availability of the daVinci Xi robot.

**Fig. 1:**
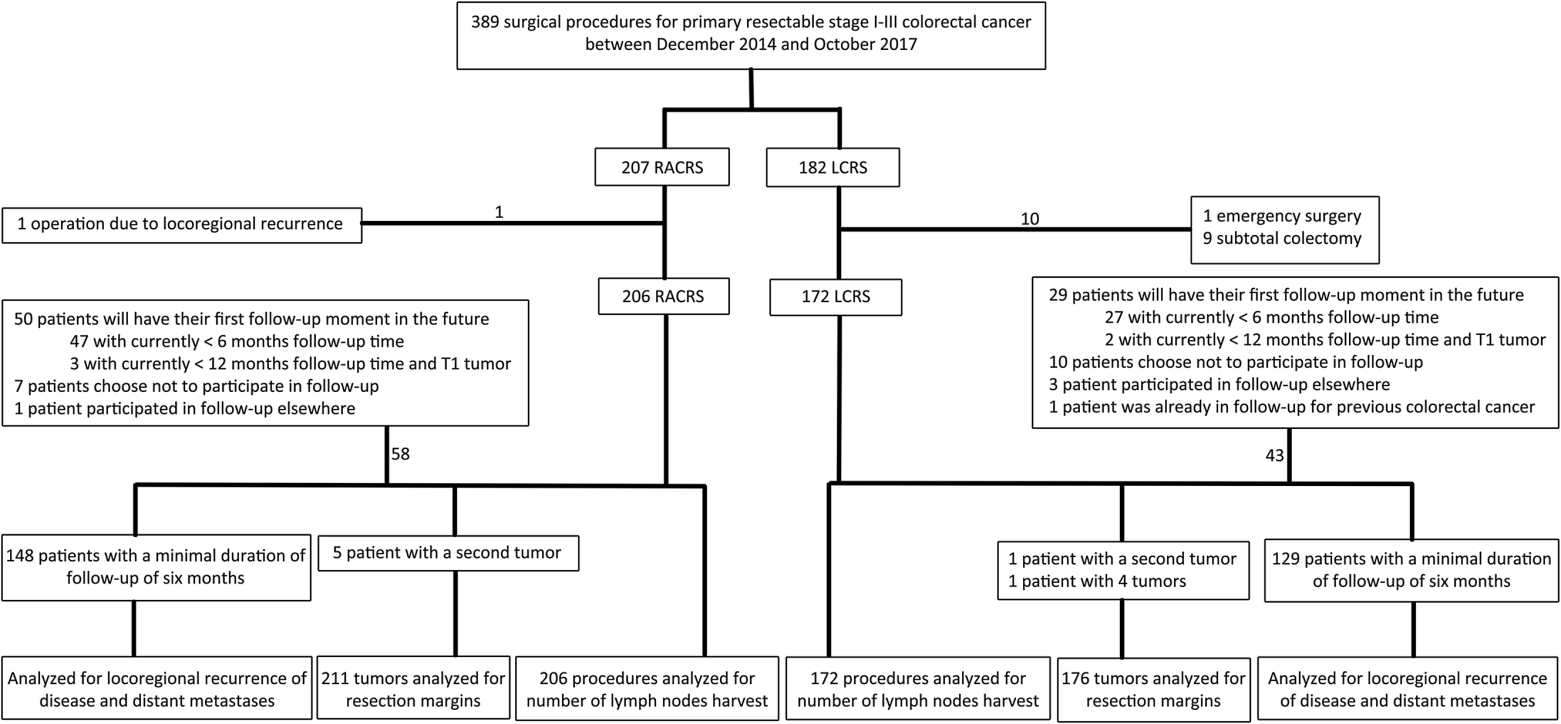
Study flowchart

Patients with a minimum follow-up of 6 months in the CWH were analyzed for oncologic outcome parameters, including locoregional recurrence rate, distant metastases, OS, and DFS. Patients with a follow-up of less than 6 months, and patients that choose to participate in follow-up elsewhere were excluded for this analysis. Figure 1 shows an overview of the patients’ population.

### Pre-operative work-up

All patients were treated in consistence with the evidence-based Dutch national guidelines 2014 [14], and were discussed in a multidisciplinary consultation, including surgeon, gastroenterologist, oncologist, radiologist, and radiotherapist, prior to and after surgery. Chest radiography and computed tomography of the liver was performed preoperatively to rule out distant metastases in lung and liver, respectively. In case of rectal cancer, magnetic resonance imaging (MRI) of the rectum was performed to determine clinical cancer stage. Patients with intermediate- and high-risk rectal cancer were treated by neoadjuvant therapy.

### Surgical procedures

The procedures were totally laparoscopic or totally robotic. Types of surgery performed for colon cancer were left hemicolectomy, right hemicolectomy, transverse colon resection, sigmoid resection, or low anterior resections. Conventional medial-to-lateral and lateral-to-medial approaches were used in accordance with the preference of individual surgeons. Surgery performed for rectal cancer was low anterior resection or abdominoperineal resection with dissection of the rectum according to total or partial mesorectal excision principles. The need for abdominoperineal resection was determined by the tumor characteristics not being separable from external sphincter or pelvic floor structures. Procedures that needed opening of the peritoneal reflection and partial mesorectal excision for radical removal of the tumor were classified as low anterior resections, independent of the tumor localization (colon or rectal).

Port placement in LCRS group was according to general standardized laparoscopic principles. In the RACRS group, a 12-mm camera port was placed at the umbilicus. In addition, three 8-mm robotic instrument ports were placed along an imaginary linear line, starting from the subxiphoidal space to the left or right iliac region. The distance between each port was at least 8 cm to reduce external collisions. One additional laparoscopic trocar was placed in the contralateral flank to assist if necessary. The anastomoses were performed tension-free and either intracorporeally (circular or linear stapled) or extracorporeally (handsewn or linear stapled) dependent on the surgeons’ choice. In robot-assisted procedures, all anastomoses were performed intracorporeally. Indication for protective loop ileostomy was low colorectal anastomosis; however, the final choice was left to the decision of the surgeon.

The daVinci Xi Surgical System (Intuitive Surgical, Sunnyvale, California, United States) was used for all robot-assisted procedures.

### Post-operative care and follow-up

Post-operative care was performed consistent with the early recovery after surgery (ERAS) protocol [15].

Follow-up moments were 6, 12, 18, 24, and 36 months after surgery with blood tests and imaging according to the evidence-based Dutch national guidelines 2014 [14]. After 1 year, a colonoscopy was performed in all patients. Patients with only T1 cancer and no lymph nodes involved received a less intensive follow-up of at least colonoscopy 1 year after surgery.

### Statistical analysis

The *X*^2^ test was used to compare categorical variables and the independent-samples T test was used to compare continuous variables. For comparison of locoregional recurrence of disease, distant metastases, DFS, and OS, the Kaplan–Meier method was used and its results were compared with the log-rank test. All data were analyzed using IBM SPSS statistics 24. *P* values of less than 0.05 were considered significant.

## Results

In total, 378 surgical procedures for stage I–III colorectal cancer were included. Two-hundred and six RACRS (129 (63%) colon and 77 (37%) rectal) and 172 LCRS (138 (80%) colon and 34 (20%) rectal) procedures were analyzed.

Baseline patient characteristics did not differ between RACRS and LCRS groups (Table 1), and this similarity persists for colon and rectal cancer procedures separately. The clinical cancer stages in rectal cancer and the proportion of patients given neoadjuvant chemotherapy and/or radiotherapy were similar in both groups.

**Table 1:** Clinical characteristics

	RACRS (*n* = 206)	LCRS (*n* = 172)		*P* value
Sex, % (*n*)				
Male	66.5% (137)	57.6% (99)	0.074	
Female	33.5% (69)	42.4% (73)		
BMI, % (*n*)			0.407	
< 18	1.0% (2)	2.3% (4)		
18–25	38.3% (79)	36.0% (62)		
25–30	43.2% (89)	39.0% (67)		
> 30	17.5% (36)	22.7% (39)		
Age, mean ± SD	68.32 ± 10.02	70.11 ± 9.96	0.083	
ASA grade, % (*n*)			0.136	
ASA 1	23.3% (48)	15.7% (27)		
ASA 2	56.8% (117)	57.6% (99)		
ASA 3	19.9% (41)	26.2% (45)		
ASA 4	0.0% (0)	0.6% (1)		
History of abdominal surgery, %(*n*)	28.2% (58)	27.9% (48)	0.548	
cTNM classification, % (*n*/*n*_total_)^a^			0.546	
I	15.8% (12/76)	13.3% (4/30)		
II	14.5% (11/76)	23.3% (7/30)		
III	69.7% (53/76)	63.3% (19/30)		
Missing data, *n*	1	4		
Neoadjuvant therapy, %(*n*/*n*_total_)^b^	63.6% (49/77)	55.9% (19/34)	0.440	
Chemotherapy	28.6% (22/77)	20.6% (7/34)	0.378	
Radiotherapy	62.3% (48/77)	55.9% (19/34)	0.522	

*BMI* body mass index, *ASA* American Society of Anesthesiologist, *cTNM* clinical tumor-node-metastases stage, *RACRS* robot-assisted colorectal surgery, *LCRS* laparoscopic colorectal surgery

^a^ Clinical cancer stage of patients with rectal cancer, based on pre-operative MRI

^b^ Rectal cancer group

For colon cancer, there were significantly more sigmoidal resections performed in RACRS group (55.8% versus 33.1%, *p* < 0.001), and significantly more right-sided (34.1% versus 49.3%, *p* = 0.012) and left-sided (4.7% versus 15.2%, *p* = 0.004) colon resections performed in LCRS group. For rectal cancer, the distribution was equal (Table 2).

**Table 2:** Distribution of procedures

	RACRS (*n* = 206)	LCRS (*n* = 172)	*P* value
Colon cancer procedures, % (*n*)	62.6% (129)	80.2% (138)	< 0.001
Sigmoidal resections	55.8% (72)	33.3% (46)	< 0.001 Ɨ
Lower anterior resections	5.4% (7)	1.4% (2)	
Right colon resections	34.1% (44)	49.3% (68)	
Left colon resections	4.7% (6)	15.2% (21)	
Transverse colon resections	0.0% (0)	0.7% (1)	
Rectal cancer procedures, % (*n*)	37.4% (77)	19.8% (34)	0.794 *
Lower anterior resections	70.1% (54)	67.6% (23)	
Abdominoperineal resections	29.9% (23)	32.4% (11)	

*RACRS* robot-assisted colorectal surgery, *LCRS*: laparoscopic colorectal surgery

Ɨ *p* value of distribution of colon cancer procedures

* *p* value of distribution of rectal cancer procedures

Approximately a third (30.6%) of the rectal tumors was located too close to the anal verge for low anterior resection (median of 1 cm, mean of 2 cm) and needed abdominoperineal resection.

### Intra-operative data

The mean operation and incision time was similar in RACRS group and LCRS group for both colon and rectal procedures, and no significant differences were identified.

Six (2.9%) RACRS procedures were converted to open surgery compared to 16 (9.3%) LCRS procedures (*p* = 0.008). There was no significant difference in the colon resections: 4 conversions versus 10 conversions in RACRS and LCRS groups (*p* = 0.129), respectively. For rectal cancer, 2 conversions in RACRS group and 6 conversions in the LCRS group were performed (*p* = 0.005).

Intra-operative complications occurred in 3 out of 206 (1.5%) of RACRS procedures and in 13 out of 172 (7.6%) of LCRS procedures (*p* = 0.003). Intra-operative complications all occurred during colon resections in the RARCS group and in 10 colon (*p* = 0.062) and 3 rectal (*p* = 0.008) resections in the LCRS group. The intra-operative data are shown in Table 3.

**Table 3:** Intra-operative data

	RACRS (*n *= 206)	LCRS (*n* = 172)	*P* value
Operation time^a^, mean ± SD	180.03 ± 46.48	181.30 ± 52.34	0.806
Colon procedures	165.73 ± 43.03	171.59 ± 46.58	0.298
Rectal procedures	205.24 ± 41.57	217.88 ± 57.19	0.254
Incision time^a^, mean ± SD	134.78 ± 43.06	135.90 ± 47.19	0.815
Colon procedures	122.20 ± 40.42	126.97 ± 41.56	0.354
Rectal procedures	156.97 ± 38.60	169.50 ± 52.39	0.169
Conversion, % (*n*)	2.9% (6)	9.3% (16)	0.008
Colon procedures, %(*n*/*n*_total_)	3.1% (4/129)	7.2% (10/138)	0.129
Rectal procedures, %(*n*/*n*_total_)	2.6% (2/77)	17.6% (6/34)	0.005
Reasons for conversion, *n*			
Tumor located in descending colon	1	1	
Mobilization splenic flexure	1		
Transversostomy^b^		1	
Adhesions	2	4	
Bleeding	1		
Large tumor	1	2	
Anesthetic grounds^c^		1	
Insufficient vision^d^		5	
Bowel injury		2	
Intra-operative complications, % (*n*)	1.5% (3)	7.6% (13)	0.003
Colon procedures, %(*n*/*n*_total_)	2.3% (3/129)	7.2% (10/138)	0.062
Rectal procedures, %(*n*/*n*_total_)	0.0% (0/77)	8.8% (3/34)	0.008
Type of complications, *n*			
Fecal spill	1	1	
Arterial bleeding	2	1	
Vaginal wall injury		1	
Serosa injury bowel		3	
Full thickness injury bowel		2	
Splenic injury		2	
Bladder injury		1	
Arterial line bleeding		1	
Asystole		1	

*RACRS* robot-assisted colorectal surgery, *LCRS* laparoscopic colorectal surgery

^a^ Time in minutes

^b^ Interfering with view and instrument control and simultaneous adhesions

^c^ Ventilation problems in Trendelenburg position

^d^ Factors leading to insufficient view: obesity (3), large greater omentum (1), narrow pelvis (2), instruments too short for abdomen (1), tumor located in colon transversum (1)

### Oncological outcome

Post-operative pathological outcomes are shown in Table 4. Pathological cancer stages and type of carcinoma did not differ between RACRS and LCRS groups.

**Table 4:** Post-operative pathological outcome

	RACRS	LCRS	*P* value
pTNM (overall), %(*n*/*n*_total_)^a^			
0^b^	1.9% (4/206)	0.0% (0/172)	0.115
I	38.8% (80/206)	33.1% (57/172)	
II	25.2% (52/206)	32.6% (56/172)	
III	34.0% (70/206)	34.3% (59/172)	
Colon cancer, % (*n*/*n*_total_)			
I	38.0% (49/129)	31.9% (44/138)	0.451
II	26.4% (34/129)	32.6% (45/138)	
III	35.7% (46/129)	35.5% (49/138)	
Rectal cancer, % (*n*/*n*_total_)			
0^b^	5.2% (4/77)	0.0% (0/34)	0.468
I	40.3% (31/77)	38.2% (13/34)	
II	23.4% (18/77)	32.4% (11/34)	
III	31.2% (24/77)	29.4% (10/34)	
Tumor type, % (*n*/*n*_total_)			
Adenocarcinoma	91.0% (187/211)	88.1% (155/176)	0.226
Mucinous carcinoma	8.1% (17/211)	8.0% (14/176)	
Signet ring cell carcinoma	0.5% (1/211)	2.3% (4/176)	
Neuroendocrine carcinoma	0.5% (1/211)	0.0% (0/176)	
Undifferentiated carcinoma	0.0% (0/211)	0.6% (1/176)	
Medullary carcinoma	0.0% (0/211)	1.1% (2/176)	
R0 overall, % (*n*/*n*_total_)	95.7% (202/211)	97.7% (172/176)	0.279
R0 Colon procedures	99.3% (133/134)	98.6% (139/141)	0.592
R0 Rectal procedures	89.6% (69/77)	94.3% (33/35)	0.421
Positive distal or proximal resection margins	0.0% (0/77)	0.0% (0/35)	1.000
CRM ≤ 1 mm	10.4% (8/77)	5.7% (2/35)	0.421
Lymph nodes harvest (overall), mean ± SD (*n*_total_)	16.24 ± 6.75 (n = 211)	17.09 ± 6.83 (n = 176)	0.226
Positive lymph nodes	1.28 ± 2.73	1.07 ± 2.08	0.415
Colon procedure	16.38 ± 5.87 (n = 134)	17.54 ± 7.33 (n = 141)	0.155
Positive lymph nodes	1.37 ± 2.92	1.05 ± 2.00	0.299
Rectal procedure	16.01 ± 8.04 (n = 77)	15.26 ± 3.78 (n = 35)	0.506
Positive lymph nodes	1.12 ± 2.38	1.15 ± 2.40	0.951

*pTNM* pathological tumor-node-metastases stage, *R0* achievement of radical margins, *CRM* circumferential resection margin, *RACRS* robot-assisted colorectal surgery, *LCRS* laparoscopic colorectal surgery

^a^ Pathological cancer stage as determined by a pathologist

^b^ complete response after neoadjuvant therapy

Five patients in RACRS group and one patient in LCRS group had a second tumor; additionally, in LCRS group one patient had 4 tumors. In consequence, there were 387 tumors analyzed for resection margins. All tumors were suspected malignancies prior to the surgery, except for one tumor in RACRS group, which was a second sigmoid tumor in the pathological specimen of a sigmoid resection.

**Fig. 2:**
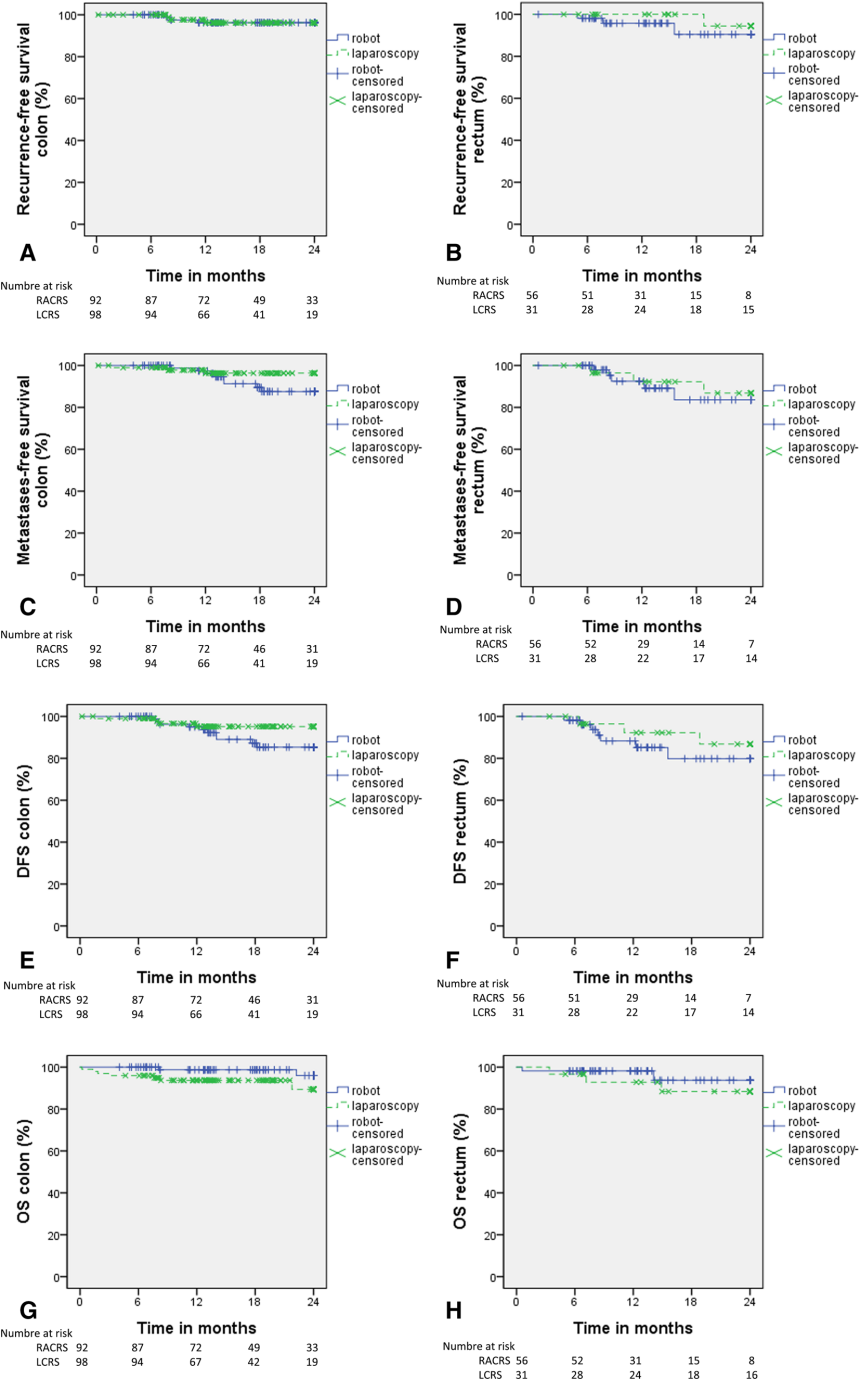
AB: 2-year recurrence-free survival. CD: 2-year metastases-free survival. EF: 2-year disease-free survival (DFS)

There were 277 patients with a minimal follow-up of 6 months and the median follow-up time was 15.3 (0.2–35.9) months. Figure 2 displays the 2-year oncological follow-up data.

#### Colon resections

Radical resection margins were achieved in 133 (99.3%) tumors in RACRS group compared to 139 (98.6%) tumors in LCRS group (*p* = 0.592). The mean harvested lymph nodes was 16.4 ± 5.9 versus 17.6 ± 7.3 (*p* = 0.155), respectively.

Recurrence of locoregional disease and distant metastases was similar for both groups in 12, 18, and 24 months after surgery. Recurrence of locoregional disease had *p* = 0. 985, *p* = 0.985, and *p* = 0.985, respectively. Distant metastases had *p* = 0.655, *p* = 0.254, and *p* = 0.170, respectively.

DFS and OS were also similar after 12, 18, and 24 months after surgery. DFS had *p* = 0.977, *p* = 0.193, and *p* = 0.132, respectively. OS had *p* = 0.116, *p* = 0.116 and *p* = 0.121, respectively.

#### Rectal resections

Radical resection margins were achieved in 69 (89.6%) tumors in RACRS group compared to 33 (94.3%) tumors (*p* = 0.421) in LCRS group. The mean harvested lymph nodes was 16.0 ± 8.0 versus 15.3 ± 3.8 (*p* = 0.506), respectively.

Recurrence of locoregional disease and distant metastases was similar for both groups in 12, 18, and 24 months after surgery. Recurrence of locoregional disease had *p* = 0.292, *p* = 0.145, and *p* = 0.419, respectively. Distant metastases had *p* = 0.940, *p* = 0.493, and *p* = 0.743, respectively.

DFS and OS were also similar after 12, 18, and 24 months after surgery. DFS had *p* = 0. 607, *p* = 0. 265, and *p* = 0. 439, respectively. OS had *p* = 0. 103, *p* = 0. 197, and *p* = 0. 197, respectively.

### Complications

Median hospital stay, re-operation rate, and 30-day mortality were similar in RACRS and LCRS groups. Furthermore, there were no differences in anastomotic leakage rates. Rates of severe complications were similar for the time periods first 30 days after surgery, first 90 days after surgery, and more than 90 days after surgery: *p* = 0.309, *p* = 0.531, and *p* = 0.167, respectively. The post-operative data are listed in Table 5.

**Table 5:** Post-operative course

	RACRS	LCRS	*P* value
Hospitalization time, median [range]^a^	5 [3–30]	5 [3–35]	
Complications < 30 days, % (*n*/*n*_total_)^b^	47.6% (98/206)	49.4% (85/172)	0.721
1	43.9% (43/98)	36.5% (31/85)	0.784
2	23.5% (23/98)	29.4% (25/85)	
3	20.4% (20/98)	20.0% (17/85)	
4	11.2% (11/98)	11.8% (10/85)	
5	1.0% (1/98)	2.4% (2/85)	
Complications < 90 days, % (*n*/*n*_total_)^b^	50.5% (95/188)	48.4% (77/159)	0.696
1	41.1% (39/95)	36.4% (28/77)	0.681
2	23.2% (22/95)	26.0% (20/77)	
3	24.2% (23/95)	20.8% (16/77)	
4	10.5% (10/95)	13.0% (10/77)	
5	1.1% (1/95)	3.9% (3/77)	
Complications > 90 days, % (*n*/*n*_total_)^b^	12.9% (19/147)	10.9% (14/128)	0.613
1	47.4% (9/19)	71.4% (10/14)	0.182
2	5.3% (1/19)	7.1% (1/14)	
3	47.4% (9/19)	14.3% (2/14)	
4	0.0% (0/19)	7.1% (1/14)	
Anastomotic leakage rate, % (*n*/*n*_total_)	4.1% (7/170)	5.2% (8/153)	0.636
After colon surgery	1.6% (2/124)	4.5% (6/133)	0.181
After rectal surgery	10.9% (5/46)	10.0% (2/20)	0.916
Re-operation rate, % (*n*/*n*_total_)	10.2% (21/206)	14.0% (24/172)	0.261
After colon surgery	7.8% (10/129)	11.6% (16/138)	0.290
After rectal surgery	14.3% (11/77)	23.5% (8/34)	0.233
30-day mortality, % (*n*/*n*_total_)	0.5% (1/206)	1.2% (2/172)	0.460
After colon surgery	0.0% (0/129)	1.4% (2/138)	0.170
After rectal surgery	1.3% (1/77)	0.0% (0/34)	0.504

^a^ Hospitalization time in days

^b^ Scored with Clavien–Dindo classification

## Discussion

In this longitudinal prospective cohort study in colorectal cancer patients, it is demonstrated that radical margins as well as lymph nodes could be achieved with RACRS as adequate as with LCRS and that locoregional recurrence rates were similar during the first 24 months’ follow-up. RACRS had less conversions and less intra-operative complications as compared to LCRS.

To our best knowledge, this study is the first cohort study comparing RACRS performed by the latest version of daVinci Intuitive Surgical system (daVinci Xi) with conventional LCRS. Several retrospective studies with mainly small sample sizes have evaluated the short-term outcomes of robot-assisted surgery for colon cancer [11, 16–21] and rectal cancer [9, 10, 16, 17, 22–28,]. The first results were promising. However, well-designed studies evaluating oncological outcome have rarely been conducted.

Radical resection margins and number of lymph nodes harvest are important factors in the assessment of quality of surgery. Excision of malignancies with appropriate tumor-free margins is considered curative. In rectal cancer, circumferential resection margin (CRM) involvement is a predictor of poor prognosis, increasing the risk of locoregional recurrence of disease by 3.5 times [29]. In the current study, no significant differences were shown in resection margins and number of lymph nodes harvest for both colon and rectal cancer procedures between RACRS and LCRS. This finding is in line with previous literature concerning colon [11] and rectal [22–25, 28] cancer for RACRS and LCRS.

In terms of recurrence of disease, regular follow-up showed no significant difference between RACRS and LCRS groups in the first 24 months after surgery. Local recurrence rate in colon cancer was 3.8% for both RACRS and LCRS groups and in rectal cancer 9.5% versus 5.6% (*p* = 0.419), respectively. Similar to the present study, two comparative studies suggest equal locoregional recurrence rate for rectal cancer, of which one prospective analysis [28]. The other was a phase II open label prospective randomized controlled trial performed by Kim et al. [10] of 66 robotic and 73 laparoscopic procedures for cT1-3NxM0 rectal cancer. Locoregional recurrence at 3 years was 5.0% in each of the study groups. In the literature, however, studies regarding robot-assisted surgery have mainly lack of sufficient follow-up and/or sample sizes or did not analyze oncological follow-up data. There are more data available regarding the conventional laparoscopic and open colorectal surgery. The MRC CLASICC group [4] performed a randomized trial comparing 526 laparoscopic to 268 open procedures for colon and rectal cancer and showed a local recurrence rate in laparoscopic surgery group of 7.3% for colon cancer and 9.7% for rectal cancer at 36 months. In this study, 2.9% of patients had liver metastases (stage 4) which is curatively treated. A multivariate analysis by Jagoditsch [30] to determine factors influencing prognosis in radical resected colon cancer showed that pathological TNM stage had independent influence on survival, but not on recurrence. Therefore, exclusion of stage 4 in the present study may not significantly affect the oncological outcome.

An often mentioned disadvantage of RACRS is a supposed increased operation time. The majority of previously conducted research determined a prolonged operation time for RACRS compared to LCRS [10, 12, 17, 19, 22, 24]. In contrast, in the present study, a similar operation time is observed. There was no difference for the type of surgery [data not shown]. The relatively short operation time in RACRS groups could be due to the use of the more advanced daVinci Xi robot in this study compared to its predecessors in the previously mentioned studies. DaVinci Xi has a wider range of motion and the possibility of attaching the endoscope to any arm, which could make it easier to operate in multiple quadrants and narrow spaces such as the pelvis. Furthermore, previous research already compared daVinci Xi to its latest predecessor daVinci Si and noticed a significant shorter docking time and operation time for daVinci Xi in rectal cancer [31, 32]. Therefore, it seems reasonable that the operation times were shorter in RACRS group than generally observed in the literature.

Surgical safety of robot-assisted procedures is as important as the oncological safety to approve its use in colorectal cancer. Hospitalization time and complication rates were similar between RACRS and LCRS groups. A higher rate of conversion (*p* = 0.008) and intra-operative complications (*p* = 0.003) was identified in the LCRS group. In current study, reasons for conversion were mainly anatomic and tumor-specific reasons (5/6 in RACRS group and 9/16 in LCRS group). In LCRS group, 7 patients were converted due to insufficient view or complication, compared to only 1 in RACRS group. Additionally, in LCRS group, injury of nearby organs (i.e., bowel, spleen, bladder, and vaginal wall) occurred 9 times, but none of these events occurred in RACRS group. Similarity in patient characteristics and clinical and pathological cancer stages argue against a reflection of selection bias. Improved visualization and advanced control in robot-assisted surgery could have prevented occurrence of conversions and complications in RACRS group. Furthermore, improved intra-operative outcomes in robot-assisted surgery are also earlier reported. A prospective analysis performed by Baik et al. [23] of 56 robotic and 57 laparoscopic procedures for rectal cancer and a retrospective cohort study performed by Mirkin et al. [11] of 765 robotic and 14,347 laparoscopic procedures for colon cancer also reported significantly more conversion in LCRS groups.

The present study had some limitations. First, this study was not double-blinded or randomized. It was a retrospective analysis of a prospectively collected database and therefore subject to selection bias, associated with this type of study design. This resulted in a distribution of colon and rectal cancer procedures that was not similar between RACRS and LCRS group. However, patients were all operated in the same period with similar peri-operative treatment protocol. Clinical characteristics of patients, indications of operation, and pathological cancer stages were similar between RACRS and LCRS group. This similarity persists for colon and rectal cancer procedures separately. Supplementary analyses were performed to identify confounding. For intra-operative and pathological outcome, additionally, robot-assisted procedures were compared to laparoscopic procedures for colon and rectal resections separately. On the other hand, a smaller number of surgeons were experienced with RACRS procedures compared to LCRS procedures. Therefore, there was a wider range of surgeons performing LCRS, possibly making the procedure more variable with different preferences in surgical approaches. By obligation of nationally approved laparoscopic surgical skills training for all surgeons and an additional training module for the surgeons performing RACRS, an adequate, minimal level of surgical skills was established. Small differences, however, cannot be ruled out. Furthermore, the current study was started in the implementation phase of daVinci Xi in the CWH. Internal analysis showed a learning curve of 32 interventions for operation time for an individual surgeon. No learning curve effects were found regarding clinical outcome parameters. Therefore, operation time in RACRS group might be shorter than observed in the full study period, but no other biases are expected.

The median follow-up time of the present study was relatively short to make definitive comparisons of oncological follow-up data. However, oncological follow-up of robot-assisted surgery has rarely been described in the literature. Kang et al. [20] described oncological outcome in robot-assisted surgery with a median follow-up of 40 months. Unfortunately, a very limited cohort of 20 robot-assisted procedures was included. Patriti et al. [26] described oncological outcome with a mean follow-up of 12 months, but had only 29 robot-assisted procedures analyzed. The only study with a relatively large sample size of 133 robot-assisted procedures and a proper median follow-up of 58 months, describing oncological outcome, was performed by Park et al. [28]. However, this Korean study was subject to major bias because patients had to choose between laparoscopic and robot-assisted surgery themselves and due to the Korean health care system, patients had to pay an additional $6000 for choosing robotic approach. Also, only procedures for rectal cancer were analyzed. The present study provides us with the first analyses of oncological follow-up of both colon and rectal robot-assisted procedures, compared to conventional laparoscopic procedures in the same hospital. Robotic surgery is a very developing subject and has a continuous progress in innovation. In this study, we used the most advanced, most up-to-date version of robotic surgery available. However, some already available technologies on the robot such as fluorescence-guided surgery are not used. These innovations may improve the clinical outcomes in RACRS in the future.

In conclusion, RACRS is safe and feasible in the treatment of primary resectable stage I–III colorectal cancer. Oncological outcome did not differ with similar radical resection margins, lymph nodes harvested, and locoregional recurrence during 24 months after surgery compared to LCRS. Benefits of RACRS have been noticed for operative outcomes, i.e., less conversion rate and intra-operative complications. Furthermore, similar operative times have been observed for RACRS and LCRS.

Future clinical trials with daVinci Xi should be realized to confirm current evidence in favor of RACRS and the suggestions of shorter operation times. Furthermore, extended research is needed to determine whether equal oncological outcomes will last in long-term follow-up.
